# Massive and massless charge carriers in an epitaxially strained alkali metal quantum well on graphene

**DOI:** 10.1038/s41467-020-15130-1

**Published:** 2020-03-12

**Authors:** Martin Hell, Niels Ehlen, Giovanni Marini, Yannic Falke, Boris V. Senkovskiy, Charlotte Herbig, Christian Teichert, Wouter Jolie, Thomas Michely, Jose Avila, Giovanni Di Santo, Diego M. de la Torre, Luca Petaccia, Gianni Profeta, Alexander Grüneis

**Affiliations:** 1 II. Physikalisches Institut, Universität zu Köln, Zülpicher Strasse 77, 50937 Köln, Germany; 2 Department of Physical and Chemical Sciences and SPIN-CNR, University of L’Aquila, Via Vetoio 10, I-67100 Coppito, Italy; 3 Institute of Physics, Montanuniversität Leoben, Franz Josef Str. 18, 8700 Leoben, Austria; 4 Institute for Molecules and Materials, Radboud University, AJ Nijmegen, Netherlands; 5 ANTARES Beamline, Synchrotron SOLEIL & Universite Paris-Saclay, L’ Orme des Merisiers, Saint Aubin-BP 48, 91192 Gif sur Yvette Cedex, France; 6 Elettra Sincrotrone Trieste, Strada Statale 14 km 163.5, 34149 Trieste, Italy

**Keywords:** Electronic properties and materials, Electronic properties and materials, Surfaces, interfaces and thin films, Surfaces, interfaces and thin films, Nanoscale materials

## Abstract

We show that Cs intercalated bilayer graphene acts as a substrate for the growth of a strained Cs film hosting quantum well states with high electronic quality. The Cs film grows in an fcc phase with a substantially reduced lattice constant of 4.9 Å corresponding to a compressive strain of 11% compared to bulk Cs. We investigate its electronic structure using angle-resolved photoemission spectroscopy and show the coexistence of massless Dirac and massive Schrödinger charge carriers in two dimensions. Analysis of the electronic self-energy of the massive charge carriers reveals the crystallographic direction in which a two-dimensional Fermi gas is realized. Our work introduces the growth of strained metal quantum wells on intercalated Dirac matter.

## Abstract (web summary)

Cesium atoms that are grown on intercalated bilayer graphene can create an ordered epitaxial film. Here, the authors report that such a strained film can host quantum well states with high electronic quality as characterized through angle-resolved photoemission spectroscopy.

## Introduction

Van-der-Waals heteroepitaxy^1^, that is the growth of dissimilar two-dimensional (2D) materials on top of each other is a major working horse for current 2D materials science. Graphene has turned out to be an excellent substrate for van-der-Waals heteroepitaxy and the growth of transition metal dichalcogenides such as MoS_2_, MoSe_2_, or TaS_2_ has been achieved on epitaxial monolayer graphene^2–6^. Epitaxial growth of thin films of conventional materials such as simple metals on van-der-Waals substrates is complicated by the notoriously low wetability of van-der-Waals materials^7–9^. Improving the wetability of graphene for adsorbed water has been achieved by doping graphene^10^ and changing the layer under graphene^7^. Yet, the growth techniques and the characterization of hybrid structures consisting of van-der-Waals materials and metals are unexplored. Its development can have a large impact on electronic structure engineering of 2D matter and extend growth techniques that use van-der-Waals materials as substrates. Here we introduce an epitaxial growth method for the synthesis of crystalline and strained alkali-metal films on top of bilayer graphene. Electronic structure characterization of the bilayer graphene/alkali-metal film heterostructure highlights the joint occurrence of graphene-derived Dirac Fermions and alkali metal-derived Schrödinger Fermions. The strain in the alkali-metal layer is a consequence of the lattice mismatch to graphene and the relatively high ductility of alkali metals. Graphene hosts ordered layers of alkali metals either adsorbed onto^11^ or intercalated in between individual graphene sheets^12^ or between the substrate and graphene^13^. The mechanism which dictates the alkali-metal order is the Coulomb repulsion between ionized alkali atoms. For cesium (Cs) adsorbed on or intercalated underneath monolayer graphene, a perfect order in a (2 × 2) or a ($\sqrt{3}\times\,\sqrt{3}$)R30° superlattice has been observed^11,14–16^. The interaction of epitaxial bilayer graphene with Cs has revealed that, at low Cs coverages, the energetically favorable position of Cs is below the bilayer^14^. However, the structure and the electronic properties of the large Cs coverages on bilayer graphene are completely unexplored. The growth of a 2D alkali-metal film is interesting because it is one of the best realizations of a noninteracting 2D Fermi liquid which is termed 2D Fermi gas. A Fermi gas consists of noninteracting electrons, and therefore it is an important starting point for the study of Fermion systems upon switching on interactions. Other metals than the alkali metals do not realize a Fermi gas because of interactions, e.g., hybridization, electron–electron, and electron–phonon coupling, as evidenced by deviations from the parabolic free electron like band structure^17,18^. A 2D alkali-metal film has a much lower electron density than other 2D metal films and hence is expected to have less deviations from the free electron like behavior. Experimentally, the degree of electron interaction can be probed by measurement of Σ, the complex-valued self-energy function, via angle-resolved photoemission spectroscopy (ARPES). In particular, ℑΣ, the imaginary part of Σ, is proportional to the photohole scattering rate. Thus, ARPES can be used to identify a realization of a Fermi gas in a 2D alkali-metal film.

In the present work we study the interaction of epitaxial bilayer graphene with high Cs coverages by performing longer Cs evaporation onto the graphene bilayer than the previous reports^11,14,15^. In these conditions, Cs intercalates under and in between the graphene bilayer and even grows on top of bilayer as a crystalline, thin Cs film. Intercalation between the graphene layers has been reported in the literature^12,19–21^. The Cs film that grows on top adopts a highly compressed (~11%) fcc structure which is different from the Cs bcc bulk structure. This observation is reminiscent of the phase transition of many alkali metals to an fcc structure under pressure^22–26^ which has been observed for lithium^27^, potassium^28^, and rubidium^29^. Bulk Cs has been reported to maintain the bcc structure (this phase is termed Cs-I) down to 4 K^30,31^, and a bcc to fcc (Cs-II) structural transition has been observed at a pressure of 23.7 kbar^25^. The additional Cs phases termed Cs-III, Cs-IV, and Cs-V appear upon further compression^32–34^. Theoretically, ab initio methods predict that the Cs-I and Cs-II structures are degenerate to within several meV of energy and there is no consensus which is the structure of the ground state^35–37^. It has been pointed out that electronic and dynamical effects are also important for the bcc to fcc structural transition^37,38^. Moreover, the ground state structure of a thin Cs film can also be different from the bulk Cs structure. Electronic and dimensional effects on the structure highlight the usefulness of applying ARPES and ab initio calculations in tandem since the structure of a material is also encoded in its band structure. The ARPES investigation shows the coexistence of a series of parabolic Cs 6*s* derived quantum well states and a linear graphene-derived dispersion relation. The quantum well states have a very narrow energy broadening and analysis of the self-energy indicates that there are certain crystallographic directions in which the observed broadening is dominated by instrument resolution rather than quasiparticle lifetime. The vibrational structure of pristine and Cs-intercalated bilayer graphene/Ir(111) is characterized. We show how Raman spectroscopy can be used to identify epitaxial pristine and doped bilayer graphene from the position and Fano asymmetry of the Raman *G* mode of bilayer graphene.

## Results and discussion

### Characterization of epitaxial bilayer graphene

**Fig. 1:**
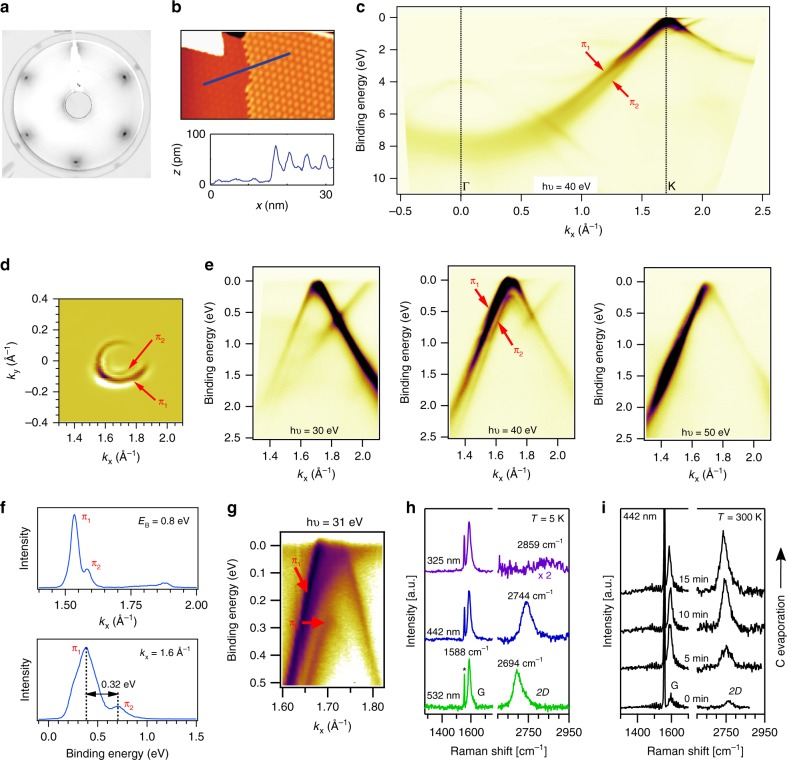
Characterization of bilayer graphene/Ir(111). **a** LEED pattern (*E* = 98 eV and *T* = 20 K) and **b** upper panel: STM topograph taken at 300 K over monolayer (left) and bilayer (right) graphene on Ir(111). Lower panel: height profile along blue line in STM topograph. **c** ARPES scan along the Γ*KM* direction of the first Brillouin zone (BZ). Labels *π*_1_ and *π*_2_ indicate the two *π* valence bands. **d** ARPES map composed of second derivative images at a constant binding energy *E*_B_ = 0.7 eV as a function of 2D wavevector. **e** ARPES scans along the Γ*K* direction for three different values of *hν* as indicated. **f** Momentum distribution curve (top) and energy distribution curve (bottom) for the *hν* = 40 eV ARPES scan of **e**. **c**–**f** are measured with circularly polarized light at *T* = 70 K. **g** ARPES scan taken with linearly *p* polarized light at *hν* = 31 eV and *T* = 15 K measured along the Γ*K* direction. **h** Ultra-high vacuum (UHV) Raman spectra of bilayer graphene grown in one step for three laser excitations as indicated. The *G* and *2D* modes are indicated and the feature at 1550 cm^−1^ denoted by ‘*' is due to O_2_ in the laser path outside the UHV chamber. **i** UHV Raman spectra of initial stages of bilayer growth during step-by-step carbon deposition onto monolayer graphene, i.e., the sample was cooled to room temperature after each C evaporation cycle.

The low energy electron diffraction (LEED) pattern of epitaxial bilayer graphene on Ir(111) is shown in Fig. 1a and shows six diffraction spots with a very weak moiré pattern (see “Methods” section for synthesis). The six diffraction spots that correspond to bilayer graphene are sharp and hence indicate negligible azimuthal disorder. The weak moiré pattern in LEED is an interesting feature that is not observed in monolayer graphene/Ir(111) which has a pronounced moiré pattern in LEED^39^. In the upper panel of Fig. 1b, we show a scanning tunneling microscopy (STM) topograph of a region with monolayer and bilayer domains visible. The lower panel of Fig. 1b, depicts a line profile of the height (*z*) variation of a scan across the domain boundary. The scan of the *z* profile in the bilayer domain reveals that the corrugation in the bilayer domain is at least a factor 3 larger compared with the monolayer domain which is the reason for the weak moiré pattern observed in LEED. We note that for ARPES and Raman, the synthesized sample had a complete bilayer coverage while for the STM measurements shown in Fig. 1b, the bilayer coverage was chosen about 50% (also see Supplementary Fig. 1). Figure 1c depicts an ARPES scan of bilayer graphene/Ir(111) which shows two *π* bands (labels *π*_1_ and *π*_2_) instead of one *π* band which is observed for monolayer graphene/Ir(111). The two observed parabolic *π* valence bands are consistent with Bernal (or AB) stacking^40^ and in agreement with earlier reports on bilayer graphene on Ir(111)^14^. An equi-energy cut around the *K* point at a binding energy *E*_B_ = 0.7 eV is shown in Fig. 1d. For clarity, we display the second derivative of the raw ARPES data. It can be seen that the equi-energy surface consists of two concentric triangularly warped contours that are centered around the *K* point corresponding to the two *π* bands of bilayer graphene. In Fig. 1e, we investigate the photon energy dependence of the ARPES spectra around the *K* point of bilayer graphene for photon energies *hν* = 30 eV, *hν* = 40 eV, and *hν* = 50 eV using circular polarized light. Both *π* bands are observed for *hν* = 40 eV whereas for *hν* = 30 eV and *hν* = 50 eV only one *π* band can be observed clearly. We attribute this variation in ARPES intensity to a matrix element effect^41^. In Fig. 1f, we show an analysis of the two *π* bands from the ARPES spectrum of bilayer graphene measured at *hν* = 40 eV. In the upper panel of Fig. 1f, a momentum dispersion curve is shown. The two indicated peaks correspond to the *π* bands. In the lower panel an energy distribution curve close to the *K* point is shown. Two peaks that are split by Δ*E* = 0.32 eV can be observed. The splitting is in reasonable agreement to tight-binding calculations with parameters fit to graphite^42^ and to ARPES experiments with bilayer graphene on SiC^43^ which yield a splitting of  ~0.4 eV. In Fig. 1g, we show an observation of the *π* band structure using *hν* = 31 eV and linearly *p* polarized light. In these conditions, both *π* bands are visible. The visibility of the second band in these conditions can be explained by the different photon energy and polarization used, as well as by the better resolution set for these measurements^44^.

The Raman spectra of the bilayer graphene sample are displayed in Fig. 1h for three laser excitation wavelengths (532, 442, and 325 nm). Raman spectroscopy has been performed after growth without exposure to ambient conditions in a home-built ultra-high vacuum (UHV) Raman system^15,45^. The observed Raman spectra of bilayer graphene in the region of the *G* and *2D* bands show strong differences from the monolayer case performed in identical conditions^15^. These differences can be used for easy identification of epitaxial bilayer graphene similar to what is routinely performed on exfoliated graphene samples. For bilayer graphene, the overall Raman intensity is increased dramatically compared with monolayer and, at particular laser energies, a strong *2D* mode appears only for bilayer graphene/Ir(111)^45^. We attribute the presence of a Raman signal for bilayer graphene to the larger corrugation of bilayer as discussed above. The corrugation increases the distance to the metal which implies a weaker screening of the electric field of the incident light. The Raman *G* band spectrum of epitaxial bilayer graphene is peaked at 1588 cm^−1^ (for *T* = 5 K). Comparing the bilayer *G* band peak maximum to the one of monolayer graphene/Ir(111) which is located at 1604 cm^−1^ (also for *T* = 5 K), we see that the *G* band of epitaxially grown bilayer graphene is downshifted by 16 cm^−1^. The downshift of the C–C vibrational frequency in bilayer graphene points to an increase in the C–C bond length and is also consistent with the larger corrugation as observed by STM. Finally we note also that there is a small shoulder around 1630 cm^−1^ which we observe only after C evaporation onto the sample and thus we relate it to the presence of bilayer graphene.

The second-order Raman mode of epitaxial bilayer graphene appears in the region of 2500–2800 cm^−1^ in Fig. 1h. Unlike for epitaxial monolayer graphene on Co^46^ and on Ir^15^ with no or very weak *2D* signal, the present epitaxial bilayer graphene sample shows a strong *2D* signal when measured with blue or green light excitation (442 and 532 nm). The intensity of the *2D* mode for ultraviolet (UV) excitation (325 nm) is much weaker in agreement with previous works^47^.

Figure 1i shows a series of UHV Raman measurements upon the growth of bilayer graphene with stepwise C evaporation onto a monolayer graphene sample. In this experiment we have deposited C onto a heated monolayer graphene sample for a time as indicated in Fig. 1i. After each C deposition, the sample was cooled to room temperature and a Raman spectrum was taken followed by the next C deposition. The difference of this method to the method used in Fig. 1h is that the samples shown in Fig. 1h are made by one step C deposition. This series highlights that the *G* band and *2D* band intensities and lineshapes are a highly nonlinear function of the bilayer coverage: already at low bilayer coverages we start to observe the *G* and *2D* Raman modes. Thus, UHV Raman spectroscopy is useful to characterize the growth of epitaxial bilayer graphene on Ir(111) in situ as a quick alternative to more demanding methods such as ARPES or STM.

### Cesium-derived quantum well states

**Fig. 2:**
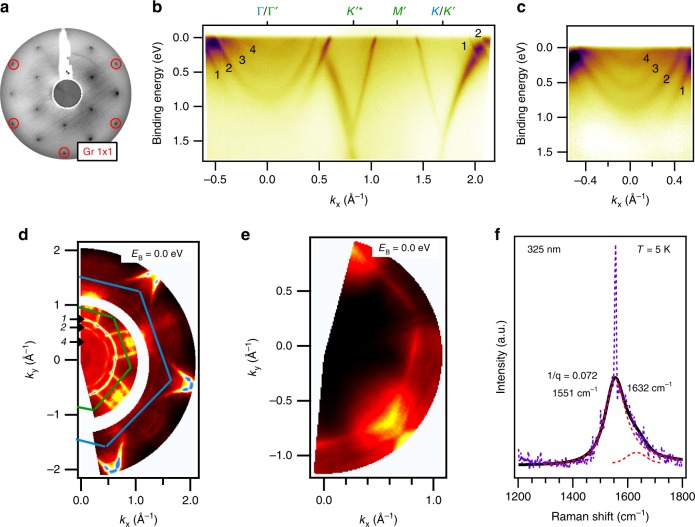
Characterization of bilayer graphene/Ir(111) after evaporation of Cs. **a** LEED pattern (*E* = 98 eV and *T* = 20 K) and **b** ARPES scan (*hν* = 31 eV and *T* = 17 K) of bilayer graphene/Ir(111) with Cs evaporated onto it. The quantum well states are labeled by 1–4. **c** Zoom-in into the region around Γ that shows four parabolic Cs quantum well states (1–4). **d** ARPES map at the Fermi energy (*E*_B_ = 0) of Cs-functionalized bilayer graphene. The quantum well states 1, 2, and 4 are indicated. The blue and the green partial hexagons denote the original and the zone-folded BZ, respectively. **e** Map as in **d** but before Cs deposition. **f** UHV Raman spectrum in the region of the *G* peak with a fit of the lineshape and the Fano parameter 1∕*q*. The black line is the fit to the experimental data which are in violet color and dashed. The two red dashed line profiles centered at 1551 cm^−1^ and 1632 cm^−1^ are used for the fitting. These two peaks are assigned to the graphene peak and to the feature that we observed before bilayer growth (cf. Fig. 1i and the discussion in the text), respectively.

Figure 2a depicts the LEED pattern of bilayer graphene/Ir(111) after evaporation of Cs. It can be seen that, in addition to the diffraction spots of bilayer graphene (cf. Fig. 1a) new spots due to the Cs lattice appear. Cs grows in a 2 × 2 superstructure with respect to the graphene lattice. The corrugation of bilayer graphene that we have discussed in Fig. 1a, b is significantly reduced by Cs intercalation. This is evident from previous works which measured Fourier transform scanning tunneling spectroscopy on Cs-intercalated bilayer graphene^14^. We believe that the flat substrate obtained by Cs intercalation is also important for the subsequent growth of Cs films. Figure 2b depicts an ARPES scan along the *ΓKM* direction of the original Brillouin zone (BZ). The interesting features that can be observed from the ARPES scan are (1) a large downshift of the Dirac points corresponding to an electron doping, (2) a single *π* band at the *K* point which is broadened in the $\Gamma ^{\prime} K{^{\prime} }^{* }$ (*KM*) direction, (3) a 2 × 2 zone folding of the band structure yielding a new Dirac cone at the $K{^{\prime} }^{* }$ point, and (4) a series of Cs-derived quantum well states located at the BZ center that cross the Fermi level (*E*_F_). Let us now discuss observations (1)–(4) in more detail. First, the Cs doping causes a partial charge transfer of the 6*s* electron to graphene and thus a downshift in energy of the full band structure. Second, an important difference to the ARPES spectrum before Cs doping is that in the present case we have a single *π* band while before doping we have two *π* bands (see Fig. 1b). The disappearance of one *π* band means that the out-of-plane coupling between the two graphene layers ceases. We explain this by Cs intercalation in between the bilayer which is consistent with the observed Cs 2 × 2 order which is also observed in graphite intercalation compounds^19^. Third, the zone folding of the band structure is caused by the Coulomb potential of the Cs 2 × 2 superstructure. The zone folding of the electronic structure of graphene is similar to what we have observed recently for Cs-functionalized monolayer graphene/Ir(111)^16^ and what has been reported for oxygen intercalated graphene^48^. Zone folding causes the appearance of a new BZ with new high-symmetry points that are labeled by $\Gamma ^{\prime}$, $K^{\prime}$, $K{^{\prime} }^{* }$, and $M^{\prime}$ that are indicated in Fig. 2b (see Fig. 1e of ref. ^16^ for a sketch of the zone-folded BZ). The broadening of the *π* conduction band of graphene along the $\Gamma ^{\prime} K{^{\prime} }^{* }$ (*KM*) direction is a result of covalent bonding of C to Cs and also present in the calculations that we will show later. Fourth, a series of four quantum well states with parabolic dispersion can be observed around the Γ point. Considering the linearity of graphene related bands in Fig. 2b, we observe that the dispersion of one *π* branch is nonlinear in the regions of *k* = 1.7 − 2.0 Å^−1^ and *k* = 0.5 − 0.86 Å^−1^. However, a linear dispersion is observed in the other branch, that is for the regions of *k* = 1.5 − 1.7 Å^−1^ and *k* = 0.86 − 1.0 Å^−1^. Irrespective of the existence of one nonlinear branch, Dirac Fermion behavior is retained for important physical properties such as the optical properties. These are determined by the electronic states far away from the *K* point, i.e., by states from the nonlinear bands. In the visible spectral range, graphene absorbs about 2.3% of light, independent of the photon energy up to  ~3 eV^49^. From Fig. 2b, it is clear that the Dirac crossing point appears at roughly 1.5 eV binding energy. This means that an optical transition induced by light of 3 eV energy involves states in the nonlinear segment of the band structure and yet results in an optical absorption governed by Dirac Fermions.

Figure 2c shows the cut through Γ at improved contrast and with the quantum well states labeled by 1–4. We performed a parabolic fit of the quantum well state dispersion measured by ARPES to extract the electron effective mass. From the fits we found that, starting from high binding energy, the masses of the sub bands 1–4 are equal to *m*_1_ = 1.0*m*_0_, *m*_2_ = 1.0*m*_0_, *m*_3_ = 0.8*m*_0_, and *m*_4_ = 0.6*m*_0_. Here *m*_0_ is the free electron mass. Since the bottom of the parabolic band of the quantum well state 1 has a very low ARPES intensity, a parabolic fit of this band is too unreliable to draw conclusions regarding Fermi gas behavior from it. Instead, we will evaluate the self-energy of this band later in the paper to confirm a Fermi gas behavior. The origin of the deviations from the free electron mass of quantum well states 3 and 4 could be related to zone folding of the bulk Cs band structure or the fact the Cs orbitals that make up quantum well states 3 and 4 are adjacent to graphene. Quantum well state 3 comes from Cs orbitals that belong to the first layer of Cs atoms grown on top of the bilayer and quantum well state 4 is from Cs orbitals of intercalated Cs atoms. The proximity of C and Cs orbitals can cause hybridization between them^16^ which is clearly visible from Fig. 2b. The hybridization of the C-derived *π* bands and the Cs quantum wells is clearly visible along the $\Gamma ^{\prime} K{^{\prime} }^{* }$ direction of the *π* band. Importantly, we have not been able to synthesize structures with more Cs bands than observed in Fig. 2c. Figure 2d shows a 2D ARPES intensity map at constant energy *E* = *E*_F_. In the ARPES map we observe three circular equi-energy contours around the *Γ* point that correspond to quantum well states 1, 2, and 4. The quantum well state 3 is not observable at *E*_F_ due to its weak intensity relative to the other features. The isotropic shape of the quantum well states in wavevector space highlights that these are free electron like states. For comparison, we show in Fig. 2e the map of pristine bilayer graphene taken in an identical configuration before Cs deposition. This reveals that the round features close to Γ are indeed Cs induced quantum well states and do not stem e.g., from Ir. Figure 2f depicts a UHV Raman spectrum of Cs-doped complete bilayer graphene (Cs doping was carried out after the Raman spectra shown in Fig. 1h were taken). The Raman *G* band of Cs-doped bilayer graphene has a large downshift and a Fano asymmetric shape which arises from electron doping and is in agreement to previous works on monolayer graphene^15,16^. Performing a fit with one Fano line and one line for the feature at 1632 cm^−1^ that we identified already in the pristine bilayer samples (see Fig. 1i), we find a *G* band position at 1551 cm^−1^ which can be explained by the previously established relation between doping and *G* band frequency^15^.

### Density functional theory (DFT)

We employ DFT in order to identify the structure of the Cs/bilayer graphene system. The correct structure should fulfill two criteria. First, its energy must be within k_B_*T* to the calculated global energy minimum (k_B_ is the Boltzmann constant and *T* = 300 K). Second, its band structure must be in agreement with the experimental ARPES spectra. We apply constraints regarding the possible structures that we derive from the Cs amount deposited and the Cs structure observed. We have deposited about five atomic layers of Cs onto bilayer graphene but will consider also structures with four and six Cs layers to take into account Cs loss during the intercalation and inaccuracies of the quartz microbalance. We observed a 2 × 2 Cs order with respect to graphene which enforces an in-plane Cs–Cs distance of 4.94 Å. Since we observed four Cs-derived bands, it is clear that we have at least four Cs layers. More layers are possible if their Cs atoms are fully ionized and hence their bands are above *E*_F_. Indeed, a Cs layer intercalated in between graphene and the Ir(111) substrate is fully ionized^16^ and the electronic band formed by these Cs orbitals is located above *E*_F_ and hence cannot be measured using ARPES. However, intercalation of a second Cs layer under graphene has not been reported and for Cs-intercalated monolayer graphene, excess Cs forms a layer on top of graphene rather than a second intercalant layer^16^. For the discussion of possible structures, it is convenient to adopt the following notation for the structure that consists of bilayer graphene and Cs layers which can be intercalated or adsorbed. In our notation, an *ℓmn* system represents *ℓ* Cs layers intercalated in between Ir(111) and the bottom graphene layer, *m* Cs layers intercalated in between the two graphene layers, and *n* Cs layers grown on top of bilayer graphene. All Cs layers have a 2 × 2 Cs order with respect to graphene. We have analyzed, using DFT, all possible *ℓmn* structures that fulfill the above mentioned constraints. In particular, we cast the above mentioned constraints as *ℓ* + *m* + *n* = 4,…,6, *ℓ* = 0, 1, *m* = 0,…,3, and *n* = 0,…,6. Applying these constraints results in the following 24 possible *ℓmn* structures: *ℓmn* = 004, 005, 006, 013, 014, 015, 022, 023, 024, 031, 032, 033, 103, 104, 105, 112, 113, 114, 121, 122, 123, 130, 131, and 132. We have systematically investigated the stability, the total energy and the electronic energy band structure of these *ℓmn* structures and find that the 113 phase has lowest total energy. We find that most of these structures can be excluded from being candidates that can describe the experimental results on the basis of having a too high total energy or a misfit of the electronic structure with the experiment.

**Fig. 3:**
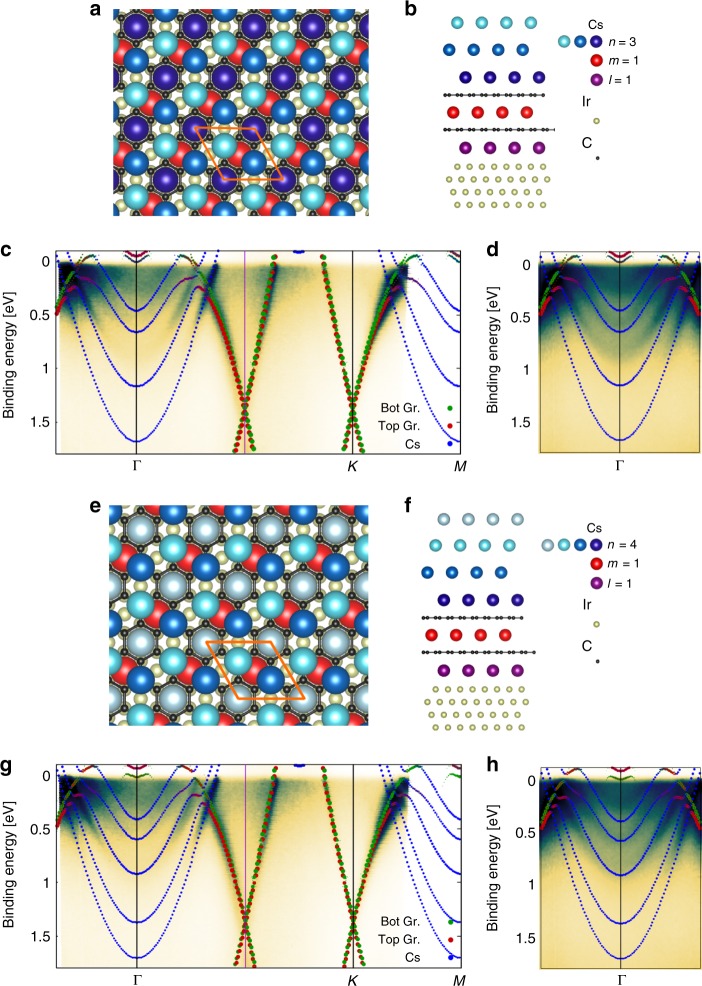
Comparison of band structure calculations to ARPES experiment. **a**, **b** *ℓmn* = 113 structural model viewed from top and from the side. The unit cell is indicated by an orange rhombus in **a**. **c** ARPES data overlaid by the DFT calculations of the 113 structure shown in **a**, **b**. **d** Region of the quantum well states overlaid with the calculation with a modified color scale to **c**. **e**, **f** *ℓmn* = 114 structural model viewed from top and from the side. **g**, **h** like in **c**, **d** but for *ℓmn* = 114.

The formation energy difference between any given *ℓmn* phase and the reference 113 configuration is defined as ${\rm{\Delta }}{E}_{X}({\mu }_{{\rm{Cs}}})\,=\,{E}_{X}\,-\,{E}_{113}\,-\,({N}_{X}^{{\rm{Cs}}}\,-\,{N}_{{\rm{1}}13}^{{\rm{Cs}}}){\mu }_{{\rm{Cs}}}$, where *μ*_Cs_ is the chemical potential of the Cs atom. The number of Cs atoms in the *X*-phase is given by ${N}_{X}^{{\rm{Cs}}}$ and in the ground state by ${N}_{{\rm{Cs}}}^{{\rm{GS}}}$. In Table 1 we report Δ*E*_*X*_ for the six structures having lowest energy, assuming Cs-rich experimental conditions (*μ*_Cs_ = *E*_Cs-Bulk_), where *E*_Cs-Bulk_ is the energy per atom in the stable Cs bulk phase. Varying *μ*_Cs_ between *E*_Cs-Bulk_ and *E*_Cs-atom_ (energy of an isolated Cs atom) in order to mimic Cs-rich and Cs-poor experimental conditions, we find that the 114 phase is favored for *μ*_Cs_ − *E*_Cs-Bulk_ > 6 meV. Figure 3a, b depicts sketches of the 113 structure. In Fig. 3c, d, we show a comparison of the experimental ARPES spectra to calculations of the 113 electronic structure which reveals reasonable agreement to the ARPES measurements. Interestingly, the 114 band structure is in better agreement with the ARPES experiment (Fig. 3e–h depict the structure and electronic structure of the 114 phase, respectively). The Supplementary Fig. 2 contains comparisions between the DFT calculations and ARPES of several *ℓmn* structures.

**Table 1:** Energy differences Δ*E*^*X*^(0) of the six lowest energy *ℓmn* structures with respect to the ground state phase 113 (Δ*E*^*X*^(0) is explained in the main text).

Structure *ℓmn*	Δ*E*^*X*^(0) (eV/unit cell)
122	0.100
121	0.100
131	0.050
112	0.035
114	0.030
113	0.000

Here *ℓmn* denotes *ℓ* Cs layers intercalated in between the graphene bilayer and the substrate, *m* Cs layers intercalated in between the bilayer, *n* Cs layers adsorbed to the graphene bilayer.

The improved agreement to the experiment is particularly true for the Cs-derived quantum well states. Since the 114 phase has five incompletely ionized Cs layers, we obtain five calculated Cs bands. This seems to be inconsistent to the experiment at first sight since the ARPES band structure shows only four Cs quantum well states. Nevertheless, a close look to the comparison of ARPES with the calculated band structures in Fig. 3g, h reveals that only four Cs bands cross *E*_F_ and that two Cs band merge with each other below *E*_F_. Since the photoemission intensity of the lowest energy Cs state is practically equal to zero around the *Γ* point, we cannot exclude the presence of a fifth Cs band in its proximity. Given the better agreement of the 114 band structure with the ARPES and the unknown experimental *μ*_Cs_ value, we conclude that the experimental structure is likely the 114 phase.

Thus, let us summarize our findings on the layer-by-layer intercalation of bilayer graphene with Cs. With increasing Cs amount, the Cs will first intercalate between the bilayer graphene and the metal followed by intercalation in between the bilayer. Finally, adsorption on top of bilayer graphene will occur. From an energetic perspective, such a hierarchy of intercalation events is reasonable because intercalated Cs has a higher binding energy than adsorbed Cs. Energetically preferred intercalation of Cs was reported also for graphene monolayer/Ir^11^ and the long-standing literature of intercalated few-layer graphene^12,20^ and graphite intercalation compounds^19^ have made a case that the intercalation of Cs in between the bilayer is not surprising. What is rather surprising in the present work is the fact that high-quality Cs quantum wells can be grown on top of such intercalated samples.

### Wetting of bilayer graphene by Cs

Let us now discuss the reason why Cs metal grows in 2D films on bilayer graphene and does not form clusters like most other metals do. Because of their low cohesive energy, alkali metals generally possess a relatively high ratio between adhesive energy on graphene and cohesive energy (*E*_a_/*E*_c_)^50,51^. This ratio is generally very low for nonalkali metals (the only notable exceptions are Nd and Sm). The low value of *E*_a_/*E*_c_ is ultimately the reason why most of the transition metals, noble metals, and rare earth metals favor a 3D growth mode when deposited on graphene as highlighted in refs. ^50,51^. For Cs however, this ratio is relatively higher than for other metals and also higher than for other alkali metals. In particular, the *E*_a_ of Cs on graphene is comparably high with respect to other alkali metals^52^ and its surface energy is among the lowest in the periodic table^53^ (nonalkali metals can have up to 10–50 times higher surface energy). In fact, the bcc (110) Cs surface energy has been experimentally measured^54^ to be  ≃0.095 Jm^−2^. Following the technique illustrated in ref. ^55^, we calculated the surface energy of the (111) surface of fcc Cs ${\gamma }_{111}^{{\mathrm{fcc}}}$ = 0.06 Jm^−2^. This is the same value reported for the hexagonal (0001) surface in ref. ^55^, which is not surprising given the similarity between the two stackings.

### Realization of a 2D Fermi gas

**Fig. 4:**
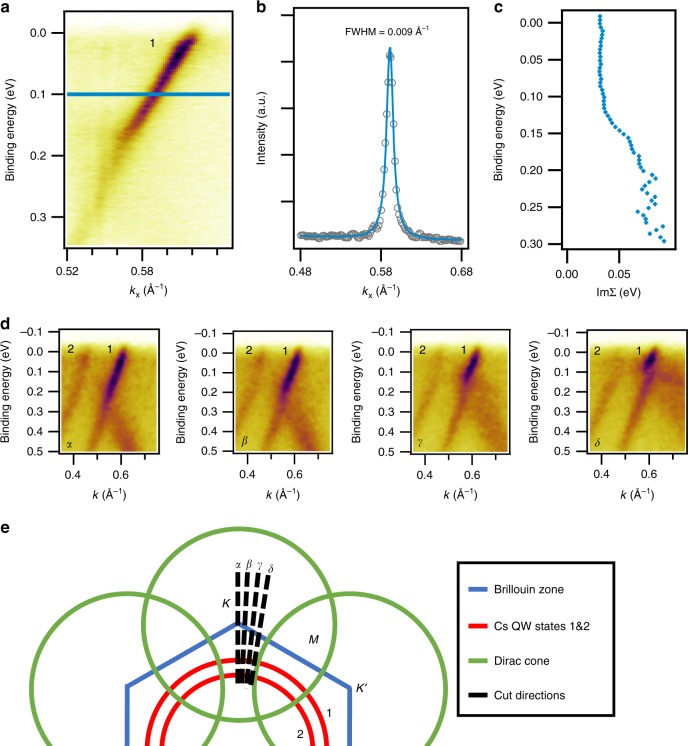
Self-energy analysis of the quantum well state. **a** High-resolution ARPES scan (*hν* = 31  eV, *T* = 20 K) of the deepest Cs quantum well state. **b** Momentum distribution curve of the cut at *E* = *E*_F_ − 0.1 eV. **c** Imaginary part of the self-energy ℑΣ(*E*) as a function of binding energy *E*. **d** Region where quantum well state bands 1 and 2 cross with the graphene band. Scans *α* − *δ* shown are radial cuts for different angles around the Γ points in steps of 3 degrees. **e** Sketch of the Fermi surface and the cuts *α* − *δ* of **d**.

The energy and wavevector dependence of ℑΣ, the imaginary part of the self-energy, of the Cs 6*s* derived quantum well states is analyzed in order to unravel the scattering mechanism. Figure 4a depicts a high-resolution ARPES scans of the quantum well state 1. Figure 4b depicts a scan through the ARPES intensity at a constant binding energy, i.e., a momentum distribution curve and reveals a full width at half maximum (FWHM) of Δ*k* = 0.009 Å^−1^ (or a FWHM of 0.16° in angular scale). Comparing this angular FWHM to the instrument resolution of 0.1° we see that the measured FWHM is strongly affected by the broadening induced by the instrument resolution. We now proceed to the analysis of the energy dependence of the imaginary part of the self-energy. This quantity is related to the FWHM Δ*k* and the Fermi velocity *v*_F_ via ℑΣ = *v*_F_Δ*k*. By investigation of the energy dependence ℑΣ(*E*) the various origins (e.g., impurity scattering, electron–phonon scattering, electron–electron scattering) of the lifetime broadening can be extracted. For example, impurity scattering gives a constant contribution to ℑΣ(*E*), electron–phonon scattering a step-like contribution^56^ and electron–electron scattering in a 2D Fermi gas has a quadratic energy dependence $\propto {E}^{{\rm{2}}}| \mathrm{ln}\,(E)|$^56–58^. Matthiessen’s rule states that these contributions to ℑΣ(*E*) are additive, and because of their distinct functional form, individual contributions can generally be extracted. For example, a detailed analysis of the strength of each of these contributions was performed, using ARPES, for the Sr_2_RuO_4_ compound^58^. The ℑΣ(*E*) for the Cs-derived quantum well state 1 (cf. Figs. 2b, c, 4a) is plotted in Fig. 4c. It can be seen that, moving away from the Fermi level, the ℑΣ(*E*) is constant up to an energy of 150 meV where it suddenly increases. The constant value of ℑΣ(*E*) for *E* between the Fermi level and binding energy of 150 meV can be taken as an indication for a very weak or absent electron–electron scattering. The large energy range where ℑΣ(*E*) is equal to a constant value does not exclude the inclusion of a component due to electron–electron scattering. Nevertheless, it puts an upper limit to the contribution of electron–electron scattering which we extract from the fit shown in Fig. 4c. Note, that ℑΣ(*E*) of conventional metals and their surface states (e.g., Cu, Mo, and Ag^59^) in a similar energy range as plotted in Fig. 4c display the typical quadratic behavior in *E* and a “kink” due to electron–phonon coupling. Qualitative comparison of the contribution of the electron–electron scattering term for the Sr_2_RuO_4_ compound^58^, the surface states of conventional metals^59^ and the present Cs metal quantum well state 1 suggests that the present system has the lowest contribution of electron–electron scattering to ℑΣ(*E*). The step-like behavior of ℑΣ around 150 meV of the Cs quantum well states can in principle have two possible origins. One is that this feature looks similar to a “kink” feature that is induced by electron–phonon coupling and the other possibility is the opening of a scattering channel for photoholes to the graphene band. The graphene *π* band and the Cs-derived quantum well state band number 1 (see Fig. 2b) cross each other in this region of the BZ and at this energy. We hence carried out a careful ARPES investigation of this feature as a function of the electron wavevector along the *α*, *β*, *γ*, and *δ* directions in the BZ (Fig. 4e). These directions correspond to angles of 0^∘^, 3^∘^, 6^∘^, and 9^∘^ with respect to the Γ*K* direction, respectively. Figure 4d reveals that the described “kink” feature appears at different energies for each slice *α*-*δ*. Inspecting its energy location, it can be seen that it appears at the crossing point between the graphene band and the band of the quantum well state 1. We hence attribute this feature to the interaction of the Cs quantum well state with the graphene band rather than to electron–phonon coupling. The sketch in Fig. 4e depicts the Fermi surface with the Cs-derived quantum well states and the graphene-derived Dirac cones. It can be seen that along *ΓK* (cut *α*) the quantum well states and the Dirac cones do not cross at *E*_F_. Therefore, the carriers along the *ΓK* direction have a weak scattering rate and this direction corresponds to the realization of a Fermi gas. For the other cuts *β*-*δ*, the crossing point between the quantum well states and the Dirac cone moves up towards the Fermi energy and scattering of quantum well states at *E*_F_ increases which can be seen from the width of the quantum well states in the ARPES experiment shown in Fig. 4d. We have also performed an analysis of the self-energy of quantum well state 2 which is shown in the Supplementary Fig. 3. We find that ℑΣ(*E*) of quantum well state 2 is significantly higher at all investigated energies *E*. In particular, for binding energies *E* < 100 meV, the ℑΣ(*E*) of quantum well state 2 is equal to 150 meV. This value has to be compared with an ℑΣ(*E*) ~30 meV for quantum well state 1 in the same energy range. The larger ℑΣ(*E*) of quantum well state 2 can be attributed to the larger scattering rate in this band due to hybridization with C states (cf. Fig. 3).

### Conclusions and outlook

In conclusion, we have prepared Cs-intercalated epitaxial bilayer graphene and used it as a substrate for the growth of epitaxially strained Cs quantum wells. This material system is a realization of a vertical heterostructure between a layered material and a metal quantum well. The well-ordered growth of Cs is understood from the improved flatness of bilayer graphene after Cs intercalation and the energy balance of the Cs surface free energy and the adhesion energy to intercalated bilayer graphene. A Cs layer forms on the intercalated bilayer graphene if the surface free energy of Cs metal is smaller than the adhesion energy to the intercalated bilayer substrate. In this case, the Cs metal can wet the intercalated bilayer graphene and an ordered thin film can grow. We have also computed the surface energy of the fcc Cs(111) surface and found that it has a very low value compared with other metals. Furthermore, alkali metals are very soft materials, and in particular Cs has a very low bulk modulus^35,37,60^ (B_0_ ≃ 2 GPa). This allows Cs to match the graphene lattice parameter with an 11% in-plane compressive strain. While we can understand from these arguments why alkali metals can wet the substrate, one would expect that a Cs layer forms equally well on monolayer graphene. Nevertheless, we have not been able to grow an ordered Cs thin film on monolayer graphene under the same conditions. We thus speculate this could be related to the adhesion energy which might be lower for the monolayer. This could be due to the fact that Cs intercalates in a $\sqrt{3}\,\times\,\sqrt{3}$ pattern under graphene which does not form a commensurate superstructure with the observed Cs 2 × 2 film that grows on top of graphene.

Performing ARPES, we investigated the electronic structure and found quantum well states with a parabolic electron energy dispersion coming from the Cs 6*s* electrons as well as a Dirac like dispersion of the *π* electrons of graphene. The electronic structure of this heterostructure thus hosts both, Schrödinger and Dirac like charge carriers. By analyzing the ARPES data we have found that the linewidth of the Cs bands is extremely narrow and ℑΣ(*E*) is constant as a function of binding energy *E* until the value of *E* which corresponds to the crossing point of the quantum well state and the Dirac cone. At the crossing point, the lifetime of the carriers decreases (revealed by an increase of ℑΣ) because of hybridization between the Cs and C bands. The sharpness of the quantum well state bands is a result of the low electron density and the constant value of ℑΣ(*E*) points towards a small contribution of the electron–electron scattering suggesting that this system is a realization of a 2D Fermi gas. Regarding further photoemission experiments, we expect that this system is suitable for laser ARPES measurements employing the commonly used *hν* = 6.05 eV. The Cs quantum well state 4 (see Fig. 2c) has a Fermi wavevector  ~0.2 Å^−1^, similarly to the Cu(111) surface state that was probed using this laser source. Thus, part of the observed ARPES spectra could also be measured by laser ARPES. Such a laser ARPES experiment would benefit from better spatial, energy and momentum resolution as compared with the synchrotron light source used here. This experiment could therefore be useful for the investigation of fine details in the quantum well states such as electron–phonon coupling and spin–orbit interactions.

We believe that the Cs/bilayer graphene heterostructure which we have synthesized can serve as a substrate to grow quantum wells made of conventional metals and semiconductors on top. The role of Cs would be crucial in such an experiment since it could provide the large adhesion energy needed so that the conventional metal or semiconductor wets the surface. The large adhesion energy could come from deposited metals that form alloys with Cs. For example, Cs forms a well-studied alloy with Au^61^ and an interesting future experiment could therefore be to grow Au on top of the Cs/bilayer graphene heterostructure. Another interesting future experiment is the study of the plasmon dispersion relation of the thin Cs film/bilayer graphene heterostructure. The plasmon dispersion can be studied using high-resolution electron energy loss spectroscopy and could reveal the presence of two distinct plasmon dispersion relations that correspond to 2D massive and massless particles. A knowledge of the plamon dispersion relations would also allow to assess the usefulness of this material for nanoplasmonics. Finally, it would be interesting to vary the alkali-metal type and hence the electron density in the hybrid structure.

## Methods

### Growth

Bilayer graphene was synthesized on Ir(111) by a two-step procedure which involves growth of monolayer graphene by chemical vapor deposition^39^ followed by C deposition via electron beam evaporation onto the monolayer graphene with the sample at a temperature of 1000 °C^14,62^. The amount of carbon deposited for the growth of the second layer, i.e., the bilayer coverage, was controlled by the evaporation time. We have investigated samples between monolayer and full bilayer coverage. The optimum growth time was determined by the appearance of two sharp *π* valence bands in the band structure that we observed using ARPES. The quality of bilayer graphene was investigated using LEED, STM, ARPES, and UHV Raman spectroscopy. The combined application of these methods corroborates the formation of AB (or Bernal) stacked bilayer graphene which is discussed in the following. Additional C 1*s* core level measurements by X-ray photoelectron spectroscopy are shown in the Supplementary Fig. 4. In the next step of the sample preparation, we evaporate Cs to a thickness of about two unit cells (corresponding to the bulk bcc structure) onto bilayer graphene at room temperature. The evaporation is calibrated by a quartz microbalance.

### Angle-resolved photoemission spectroscopy

ARPES measurements were carried out at the ANTARES beamline^63^ of the SOLEIL synchrotron in St. Aubin (France) and at the BaDElPh beamline^64^ of the Elettra synchrotron in Trieste (Italy). The pristine bilayers (Fig. 1c–f) have been measured at the ANTARES using a hemispherical MBS A1 analyzer with circularly polarized light at *T* = 70 K. One pristine scan (Fig. 1g) and all Cs-doped bilayer graphene (Figs. 2b–e, 3c, d) have been measured at BaDElPh using a Specs Phoibos 150 analyzer and linearly polarized light at temperatures between *T* = 15 K and *T* = 20 K. LEED was measured at *T* = 20 K. The Cs deposition was carried out in one step from SAES getters with the sample at room temperature. The Fermi surface maps from Fig. 2d, e have been generated from a symmetrized azimuthal sweep of the first BZ.

### UHV Raman spectroscopy

UHV Raman measurements were performed with the sample at *T* = 5 K and at *T* = 300 K in the back-scattering geometry using a commercial Raman system (Renishaw) integrated in a homebuilt optical chamber^45^. The exciting and Raman scattered light were coupled into the vacuum using a ×50 long-working distance microscope objective with an NA of  ~0.4 and a focal distance of 20.5 mm for lasers with wavelength 442 and 532 nm. For the UV laser, UV compatible optical elements have been used. The ×20 UV objective has a focal distance equal to 13 mm and an NA = 0.32. The position of the laser on the sample was checked by a camera in the laser path. All spectra have been calibrated in position to the O_2_ vibration at 1555 cm^−1^ (see ref. ^65^). The O_2_ vibration is visible in the spectra due to the laser path outside the UHV. In Fig. 1h, we have guided the laser through an Ar gas filled acrylic tube in order to minimize O_2_ Raman intensity while in Fig. 1i, the laser beam went through air explaining the larger O_2_ Raman mode intensity in Fig. 1i. Sample preparation and measurement were done in situ and the sample was never exposed to air. In particular, the deposition of Cs in UHV Raman experiments has been performed in the same manner as in the ARPES studies.

### Scanning tunneling microscopy

For STM, a sample with an only partial bilayer coverage was grown, enabling imaging of bilayer and monolayer graphene areas in a single topograph. Sample preparation and imaging was conducted in the UHV system ATHENE with a base pressure below 1 × 10^−10^ mbar. Image processing was conducted with the WSxM software^66^.

### Computational details and Cs layer stability

We perform DFT calculations with the projector augmented-wave pseudopotential method^67^, adopting PBE (GGA) exchange and correlation functional^68^. An energy cutoff of 400 eV for the plane wave basis and 14 × 14 × 1 Monkhorst-Pack grid^69^ for BZ sampling were used in order to ensure a total energy convergence of 1 meV. In our calculations of the Cs layer, we obtain a bulk bcc equilibrium lattice constant of 6.16 Å, ~2% larger than the experimental one^30^. Our results are in line with those in ref. ^37^, predicting that Cs-II (fcc) is the ground state (only 1 meV difference with respect to Cs-I). As pointed out in ref. ^38^, also the hexagonal close packed (hcp) structure, which is very similar to the fcc structure, should emerge as a competing phase at low temperature. Indeed, we find that hcp and fcc phases are energetically degenerate up to less than 1 meV. We found that the lowest energy phase contains only one Cs atom intercalated inside the graphene bilayer, while the other three stay on top of the bilayer. Other configurations, with two, three, or four Cs in between the two carbon layers are higher in energy by 0.054 eV, 0.135 eV, and 0.316 eV per unit cell, respectively. The intercalation process of one layer of Cs atoms inside the graphene bilayer lowers the energy of the system by 0.615 eV per unit cell. In our simulations, we have varied the stacking of the three Cs layers atop of bilayer graphene to find the minimum energy configuration. The three Cs layers on top can be arranged either according to the fcc(111) stacking (ABC) or alternatively according to the hcp stacking (ABA). These two configurations are similar and the energy difference between them is below 1 meV, so it is difficult to establish which one is observed experimentally. Nevertheless, the corresponding band structures are almost identical. Importantly, the in-plane lattice parameter of the Cs fcc trilayer is compressed by 11% with respect to the fcc (hcp) ground state; this, in turn, corresponds to an effective in-plane stress of 11 kbar, while the Cs–Cs out-of-plane distance is compatible with the equilibrium bulk fcc phase in the out-of-plane direction. Regarding the structure of bilayer graphene, we observe, that after the intercalation, graphene changes from AB (Bernal) to AA stacking for all investigated structures. This is consistent with previous findings on Li-intercalated graphene^70^.

The thickness-dependent stability of the Cs film grown on intercalated bilayer graphene is investigated using DFT. There are two energies that contribute to the total energy of the Cs film. One is the adsorption energy which is negative and the other one is the compression energy which is positive. We show that the adsorption of strained Cs layers on the intercalated graphene bilayer is energetically favored. Let us first consider the binding energy for one Cs atom on the Cs-intercalated graphene in the 2 × 2 unit cell, defined as Δ*E*_1_ = *E*_Cs-adsorbed_ − *E*_Cs-bulk_. Here, *E*_Cs-adsorbed_ is the total energy of the Cs atom on the graphene and *E*_Cs-bulk_ is the total energy per atom of Cs in its equilibrium bulk form. The result Δ*E*_1_ = −0.092 eV means that the adsorption of Cs onto the bilayer is energetically favored. The same quantity can also be calculated increasing the number of adsorbed Cs layers. In particular, for the trilayer, the formation energy is equal to Δ*E*_3_ = (*E*_trilayer_ − 3*E*_Cs-bulk_)∕3 = −0.04 eV. This demonstrates that the trilayer is still favored with respect to the formation of the unstrained bulk Cs. The same calculation for the case of four Cs layers (following the same fcc stacking) on top of the intercalated graphene bilayer results in a small positive value of Δ*E*_4_ = 0.005 eV. This would suggest that *n* = 3 is the limit for the Cs film thickness. Nevertheless, the analysis from the DFT section suggests that the 114 phase with *n* = 4 is a possibility. The value of Δ*E*_4_ is small compared with the energy corresponding to room temperature and the experimental *μ*_Cs_ value may be higher than *E*_Cs-bulk_. Moreover, the calculation does not include external effects such as the film morphology which might alter the value of the computed energies. It is therefore likely that systems up to *n* = 4 Cs layers can be stabilized.

## Supplementary information

Supplementary Information

## Data Availability

The datasets generated during and/or analyzed during the current study are available from the corresponding author on reasonable request.
