# Supplementary material for: Massive and massless charge carriers in an epitaxially strained alkali metal quantum well on graphene
Source: Nat Commun. 2020 Mar 12;11:1340. doi: 10.1038/s41467-020-15130-1 (PMC7067783; doi:10.1038/s41467-020-15130-1)
Supplement: Supplementary file 1 — Supplementary Information [file 41467_2020_15130_MOESM1_ESM.pdf]

Supplementary information for: Coexistence of massive and massless charge carriers in an epitaxially strained alkali metal quantum well on graphene

M. Hell[1], N. Ehlen[1], G. Marini[2], Y. Falke[1], B.V. Senkovskiy[1], C. Herbig[1], C. Teichert[1,3], W. Jolie[1,4], T. Michely[1], J. Avila[5], G. Di Santo[6], D.M. de la Torre[1], L. Petaccia[6], G. Profeta[2], A. Grüneis[1]

[1]*II. Physikalisches Institut, Universität zu Köln, Zülpicher Strasse 77, 50937 Köln, Germany*

[2]*Department of Physical and Chemical Sciences and SPIN-CNR, University of L'Aquila , Via Vetoio 10, I-67100 Coppito, Italy*

[3]*Institute of Physics, Montanuniversität Leoben, Franz Josef Str. 18, 8700 Leoben, Austria*

[4]*Institute for Molecules and Materials, Radboud University, AJ Nijmegen, Netherlands*

[5]*ANTARES Beamline, Synchrotron SOLEIL & Universite Paris-Saclay, L' Orme des Merisiers, Saint Aubin-BP 48, 91192 Gif sur Yvette Cedex, France*

[6]*Elettra Sincrotrone Trieste, Strada Statale 14 km 163.5, 34149 Trieste, Italy*

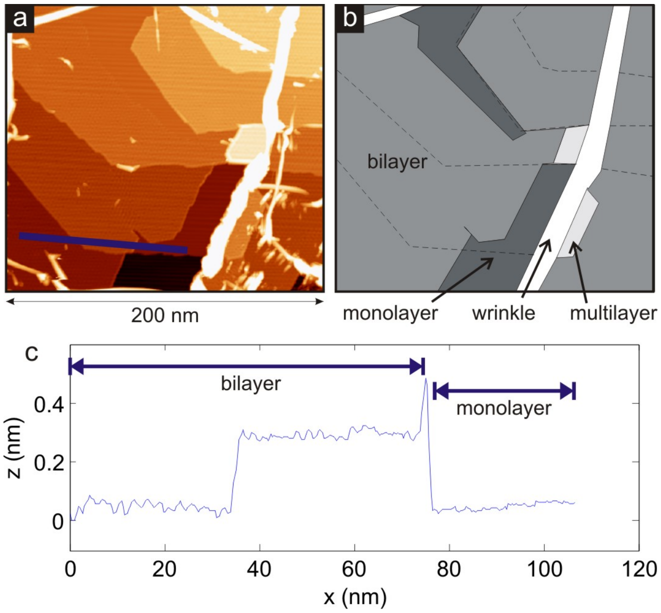

Supplementary Figure 1: STM across a region with monolayer and bilayer graphene present. (a) STM image of bilayer graphene on Ir(111). (b) Schematic representation of (a): step edges of the Ir(111) substrate are marked with dashed lines. Thick white lines denote wrinkles in the graphene layers. From dark to light gray: monolayer, bilayer and multilayer graphene.(c) Line cut along the blue line defined in (a). Monolayer and bilayer regions are identified based on the varying corrugation, which is large (small) for bilayer (monolayer) graphene.

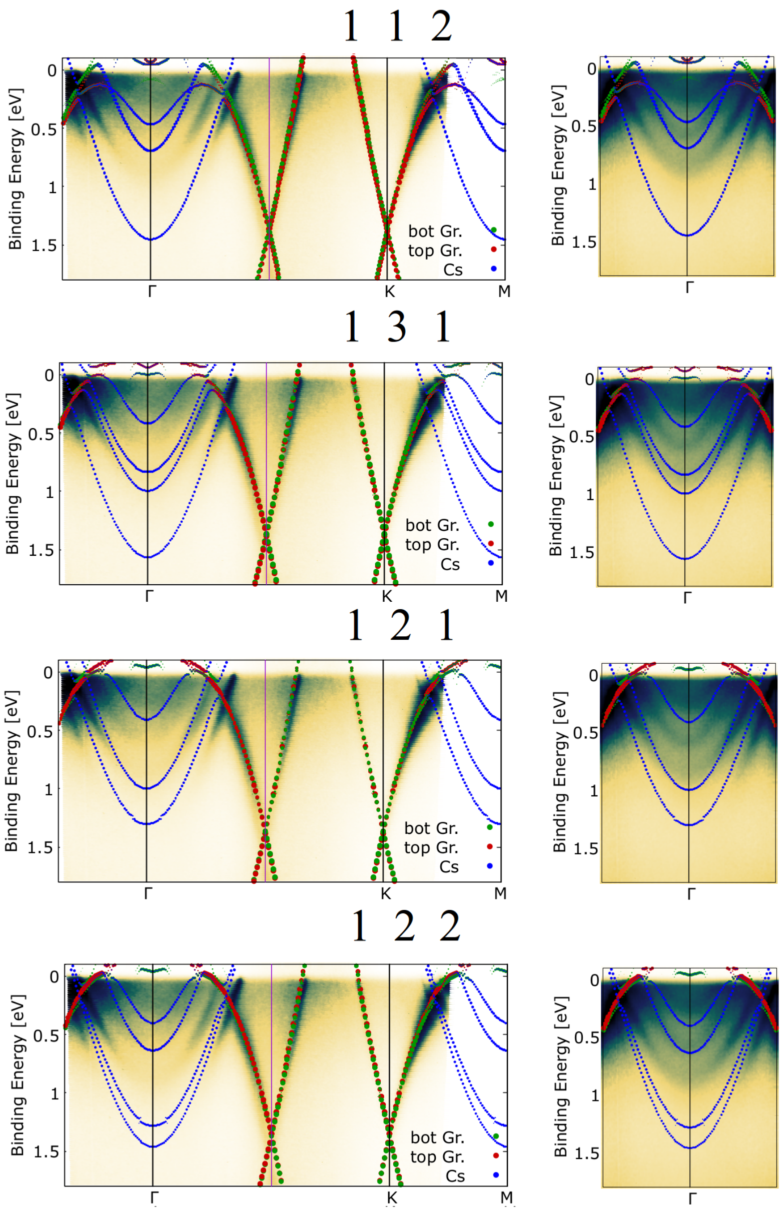

Supplementary Figure 2: ARPES spectra of Cs quantum wells grown on intercalated graphene. Comparison of theoretical calculation of various ℓmn structures to ARPES data of the Cs quantum well and graphene electronic structure.

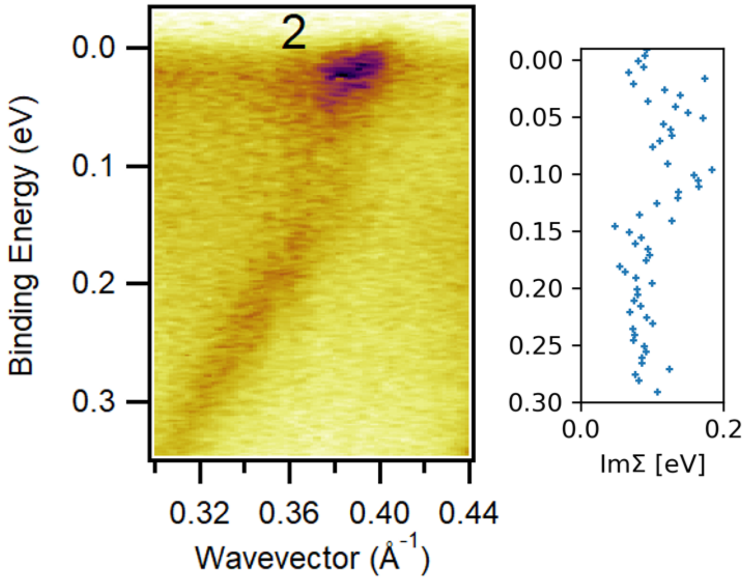

Supplementary Figure 3: Self-energy analysis of ARPES data. Analysis of $\Im\Sigma(E)$ of quantum well state 2.

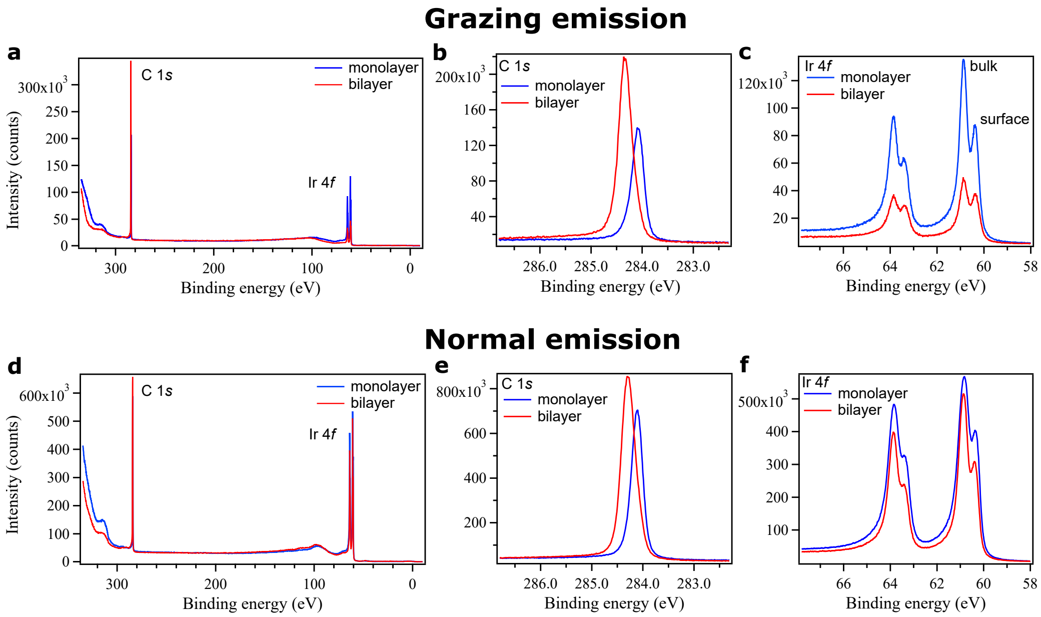

Supplementary Figure 4: XPS spectra of monolayer graphene and bilayer graphene. Upper panel: XPS spectra of monolayer graphene (blue) and bilayer graphene (red) taken in grazing emission (60° relative to the surface normal); (a) overview spectrum, (b) C1s spectrum and (c) Ir4f spectrum. Lower panel (d-f): same as in the upper panel but the spectra are taken in normal emission. All spectra are taken at a photon energ $h\nu$=370eV.
